# Effect of Ti Transition Layer Thickness on the Structure, Mechanical and Adhesion Properties of Ti-DLC Coatings on Aluminum Alloys

**DOI:** 10.3390/ma11091742

**Published:** 2018-09-16

**Authors:** Hongshuai Cao, Fugang Qi, Xiaoping Ouyang, Nie Zhao, Yun Zhou, Beibei Li, Wenzhong Luo, Bin Liao, Jun Luo

**Affiliations:** 1 School of Materials Science and Engineering, Xiangtan University, Xiangtan 411105, China; caohongshuai@aliyun.com (H.C.); zhaonie@xtu.edu.cn (N.Z.); zhouyun720@126.com (Y.Z.); libeibei06@163.com (B.L.); lwz_luowenzhong@yeah.net (W.L.); 2 College of Nuclear Science and Technology, Beijing Normal University, Beijing 100875, China; liaobingz@bnu.edu.cn (B.L.); luojun10823@126.com (J.L.)

**Keywords:** Ti-DLC coatings, FCVA, Ti transition layer, Raman, XPS, nanoindentation

## Abstract

Multilayers of Ti doped diamond-like carbon (Ti-DLC) coatings were deposited on aluminum alloys by filtered cathodic vacuum arc (FCVA) technology using C_2_H_2_ as a reactive gas. The effect of different Ti transition layer thicknesses on the structure, mechanical and adhesion properties of the coatings, was investigated by scanning electron microscopy (SEM), Raman spectroscopy, X-ray photoelectron spectroscopy (XPS), nanoindentation and a scratch tester. The results showed that the Ti transition layer could improve interfacial transition between the coating and the substrate, which was beneficial in obtaining excellent adhesion of the coatings. The Ti transition layer thickness had no significant influence on the composition and structure of the coatings, whereas it affected the distortion of the sp^2^-C bond angle and length. Nanoindentation and scratch test results indicated that the mechanical and adhesion properties of the Ti-DLC coatings depended on the Ti transition layer thickness. The Ti transition layer proved favorable in decreasing the residual compressive stress of the coating. As the Ti transition layer thickness increased, the hardness value of the coating gradually decreased. However, its elastic modulus and adhesion exhibited an initial decrease followed by an increasing fluctuation. Among them, the Ti-DLC coating with a Ti transition layer thickness of 1.1 μm exhibited superior mechanical properties.

## 1. Introduction

Aluminum (Al) alloys have wide applications in the aviation, aerospace, electronics, automobile and other industries due to advantages such as low density, high specific strength and excellent corrosion resistance [1,2,3]. However, the disadvantages of low hardness and the poor wear resistance performance of Al alloys often limit their direct usage in many areas of engineering. The surface properties of Al alloys can be improved by depositing diamond-like carbon (DLC) coatings with high hardness, low friction and excellent wear resistance [4,5,6,7,8,9]. However, when DLC coatings are deposited on Al alloys, DLC coatings have poor adhesion as a result of a serious mismatch between the thermal expansion coefficient and the mechanical properties between the DLC coatings and the Al alloys. The coatings peel off easily from substrates, which limits their service life.

In prior reports, efforts were taken to overcome the shortcomings of poor adhesion of DLC coatings. One of the common methods is to dope metal elements in DLC coatings which, in particular, offer high mechanical and tribological properties. As reported by Cicek et al. [10], good adhesion and tribological properties were obtained by depositing Ti:Ta co-doped DLC coatings on Ti6Al4V and M2 substrates. Ma et al. [11] observed that in addition to improving hardness and tribological properties, the adhesion was enhanced by adding Ti and Zr into the DLC coating on NiTi alloys. This report agrees with the views of Jo et al. [12], that Ti-DLC coatings exhibited excellent adhesion on Ti substrates. However, it is impossible to obtain excellent adhesion property by only introducing metal elements into DLC coatings, owing to the mismatch in the thermal expansion coefficient and the mechanical properties of the DLC coatings and the Al alloys. 

Furthermore, another strategy for effectively improving the adhesion of the DLC coating is to add transition or buffer layers, such as Ti, Cr, Al, Si, TiC, TiN, complex Ti/TiN, Ti/TiC/TiN, AlN/Ti/TiN, and so on [13,14,15,16]. Hee et al. [17] added a Cr buffer layer between the DLC coating and Al alloy to form a Cr/DLC coating structure, which showed excellent adhesion, high hardness and better flexibility properties. The transition layers not only effect adhesion, but can also have an influence on the structure and properties of the coatings. Liu et al. [18] studied the interfacial reaction of the composited coatings, and found that *k*-Al_2_O_3_ was formed in the coating with the bonding layer, but α-Al_2_O_3_ was formed without the bonding layer. As reported by Liao et al. [19], the Ti transition layer thickness strongly affected the structure and thickness of the gradient AlN/Ti/DLC coating, and the Ti transition layer had an optimum thickness. Bull et al. [20] believed that the adhesion of coatings decreased with the increasing of thickness and number of layers by studying failure mode and the relationship between the thickness, layer number and bonding performance, while the reason for coating failure was not studied in detail. Lee et al. [21] deposited W and WC as a transition layer (21–84 nm) and found that the adhesion property was strongly dependent on the thickness of the W layer. However, the thickness of the selected W transition layer was relatively thin, which was difficult to control during testing and not applicable for the practical industrial applications. 

In recent years, some researchers have begun to explore the possible combined effects of doping elements and adding transition layers, so as to effectively improve the adhesion between the DLC coatings and Al alloys [22,23,24]. However, the effect of the transition layer thickness on the structure, mechanical and adhesion properties is still unclear, which is of great significance for the engineering application of the DLC coating on the Al alloy. Hence, in this paper, we deposited multiplayers Ti-DLC coatings with different Ti transition layer thickness on Al alloys by filtered cathodic vacuum arc (FCVA) technology, and studied the effect of the Ti transition layer thickness on the structure and mechanical properties of the Ti-DLC coatings to obtain an optimum Ti transition layer thickness.

## 2. Experimental Details

### 2.1. Ti-DLC Coating Deposition

**Figure 1:**
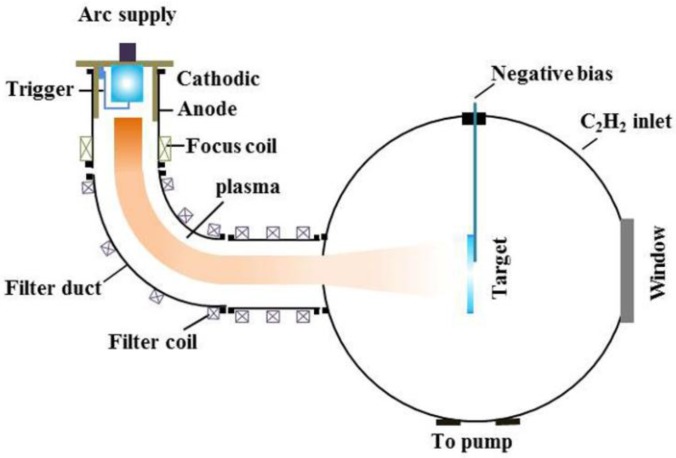
The schematic diagram of the FCVA (filtered cathodic vacuum arc) deposition system.

Al alloy samples (Al 67.7%, Si 26.7%, Cu 2.3%, and Ni 1.4 %) with the dimensions of 30 × 30 × 5 mm^3^ were used as the substrate in this study. Each substrate was polished and ultrasonically cleaned in ethanol and acetone for 20 min. The Ti-DLC coating was deposited on the substrate by FCVA technology. A schematic diagram of the deposition system is shown in Figure 1. Prior to deposition, the vacuum chamber was evacuated to a base pressure of 4 × 10^−4^ Pa. A 100 mm diameter and 45 mm thick titanium plate of 99.99% purity was used as the cathodic arc source to produce Ti plasma passing 2 kW arc power. The plasma was then guided into the processing chamber by two electromagnetic fields through a 90° duct and a 180° duct. The ducts were wrapped with solenoid coils on the atmospheric side to center the plasma along the axis of ducts. Therefore, unwanted neutral particles and macro-particles were removed by the 90° bend duct and the 180° straight duct due to the Lorentz force having no influence on them. When the C_2_H_2_ of 99.8% purity was injected in the vacuum chamber and collided with the ionized Ti plasma, new cations were formed which were available for the deposition of Ti-DLC coatings. The Ti transition layer, Ti buffer layer and Ti-DLC layer, were sequentially deposited on the substrate to form the Al alloy/Ti transition layer/Ti-DLC layer/Ti buffer layer/Ti-DLC layer coating structure. For all the experiments, these selected optimal deposition parameters were kept the same except for the different Ti transition layer deposition time *T* (min), such as −300 V for substrate bias, 80 sccm for C_2_H_2_ flow rate, duty cycle of 90%, 2.0 A and 3.5 A for filter coil currents of the 90° duct and the 180° duct, and 3 min and 10 min for the deposition time of the Ti buffer layer and the Ti-DLC layer.

### 2.2. Ti-DLC Coating Characterization

The surface and cross-sectional morphology of the coatings were analyzed using a field emission scanning electron microscope (FESEM; Nova NanoSEM 230, FEI, Hillsboro, OR, USA) equipped with an Oxford X-Max20 energy disperse spectroscopy (EDS, Oxford Instruments, Oxford, UK) system. The Ti transition layer thickness *t*_0_ (μm) was measured from the cross-sectional SEM images, and the results are shown in Table 1. The composition and structure of the coatings were investigated by EDS, Raman spectroscopy (InVia, Renishaw, London, UK), and X-Ray photoelectron spectroscopy (XPS, ECSALAB 250, Thermo Fisher Scientific, Waltham, MA, USA). Raman analysis was performed employing a laser wavelength of 532 nm in the range of 1000 to 2000 cm^−1^. The X-Ray photoelectron spectroscopy of an Al X-ray source (characteristic energy: 1.4867 keV) at a pass energy of 160 eV was used to determine the chemical bond structure of the coating before Ar^+^ erosion. 

The thickness *t_f_* (μm) of the coatings was measured by a Talysurf 5P-120 surface morphology device manufactured by Taylor Hobson, Leicester, United Kingdom. The residual stress was calculated by the change in the radius of curvature of the Al alloy substrate before and after deposition. The Stoney formula was used to estimate the residual stress which is given as:

(1) $$\sigma =\frac{E_{s}t_{s}^{2}}{6(1-v_{s})t_{f}}(\frac{1}{R_{n}}-\frac{1}{R_{0}})$$

where *E_s_*, *v_s_*, *t_s_* and *t_f_* are Young’s elastic modulus (GPa), Poisson’s radio, thickness of the Al alloy substrate and thickness of the coating, respectively.

The hardness and elastic modulus of the coatings were measured by nanoindenter equipment (TI-900 Triboindenter, Hysitron, MN, USA). In order to reduce the influence of the surface roughness and the Al alloy substrate, all tests were carried out using a Berkovich diamond tip and the maximum indentation depth was limited to 600 nm (5–10% of the total coating thickness) in continuous stiffness measurement mode. The hardness and elastic modulus values of the coatings were obtained by averaging five measurements at randomly chosen positions. The adhesion of the coatings was tested by a scratch tester (Revetest® RST^3^, Anton Pear GmbH, Shanghai, China). The scratch test was performed on the surface of the coating using a Rockwell C diamond tip (cone angle 120°) with a maximum applied load of 50 N, a loading rate of 10 N/mm and a scratch length of 4 mm. 

## 3. Results and Discussion

### 3.1. Morphology Analysis of Ti-DLC Coating

**Figure 2:**
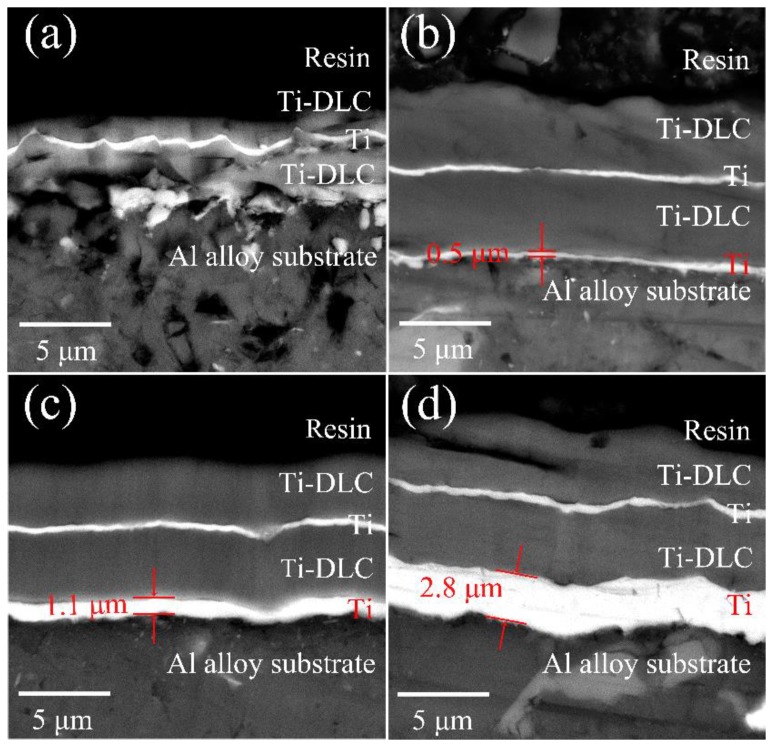
SEM cross-sectional image of the Ti-DLC coatings with Ti transition layer deposition time (*T*): (**a**) 0 min, (**b**) 5 min, (**c**) 10 min, and (**d**) 20 min.

**Figure 3:**
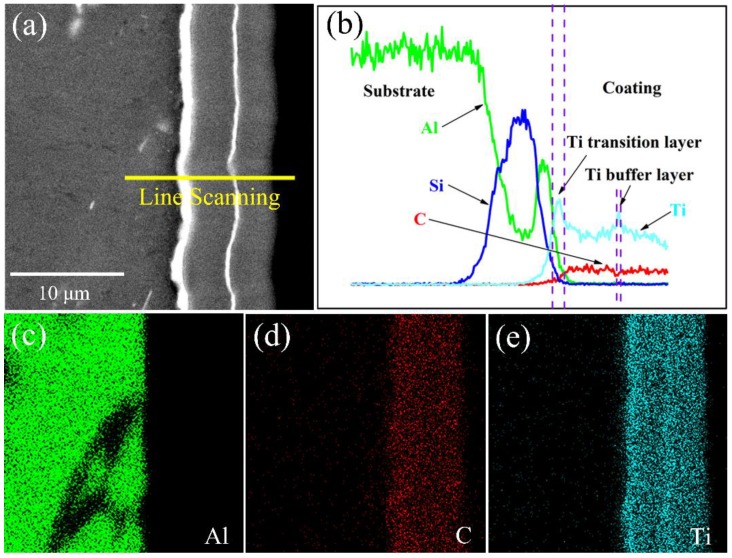
SEM cross-sectional image and EDS results of Ti-DLC coating with Ti transition layer thickness (*t*_0_) 1.1 μm: (**a**) SEM image, (**b**) EDS line scanning, and (**c**–**e**) EDS maps.

Figure 2 shows the SEM cross-sectional morphology of the Ti-DLC coatings with different Ti transition layer deposition time (*T*). The Ti layer and the Ti-DLC layer can be clearly delineated on the Al alloy. When *T* increases from 0 to 20 min, the Ti transition layer thickness *t*_0_ is about 0, 0.5, 1.1 and 2.8 μm, respectively. There is a positive correlation between the *t*_0_ and *T*, that is, the *t*_0_ increases linearly with the linear increases of *T*, indicating that the Ti transition layer can be obtained efficiently and stably by FCVA. Except for sample 1, the thickness of the Ti buffer layer and the Ti-DLC layer for all samples are about 0.5 and 4.2 μm, respectively. In addition, the overall thickness of all samples is about 10 μm except that sample 1 is about 5 μm, as shown in Table 1. Therefore, it can be preliminarily concluded that the Ti transition layer is beneficial to the growth rate of the Ti-DLC coating. Some inhomogeneity and defects, which were probably formed during grinding and polishing, can be observed in the SEM cross-section images given. In order to further analyze the chemical composition of the Ti-DLC coating, EDS maps are given by scanning along the specified line and cross-section (Figure 3). There is a smooth and clean cross section without an abrupt interface, indicating excellent adhesion properties of the Ti-DLC coating. Figure 3b shows a compact structure of the Ti-DLC coating with the Ti transition layer/Ti-DLC layer/Ti buffer/Ti-DLC layer, which is consistent with the experimental design. Typically, from left to right of the scan, the Ti element in the Ti layer first increases and then decreases in the Ti-DLC layer. A sudden rise in the abundance of Al and Si is also observed in the vicinity of substrates. Furthermore, mutual diffusion of Ti and C elements at the layer inter interfacial boundary of the substrate, the Ti layer and the Ti-DLC layer, is clearly recognized (Figure 3b–e). This diffusion results in a distribution gradient effect near the interface of the layer and serves to enhance the interfacial adhesion of the coating [25].

**Figure 4:**
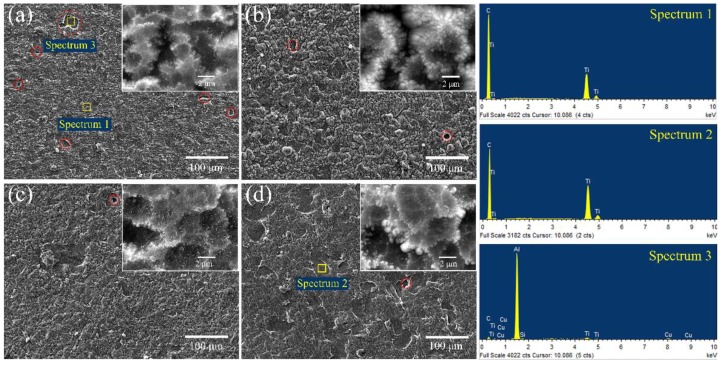
Surface SEM morphology of Ti-DLC coatings with Ti transition layer thickness (*t*_0_): (**a**) 0 μm, (**b**) 0.5 μm, (**c**) 1.1 μm and (**d**) 2.8 μm.

The surface morphology of the Ti-DLC coatings is presented in Figure 4. The surface of the as-deposited coatings are comparatively dense and uniform, and round particles are observed for all samples, which is common for FCVA technology. There are many protuberances shown as hills in the Ti-DLC coating, which increase the surface roughness. In general, the morphology of the coating depends on many factors, such as the preparation methods, deposit material properties and substrates, etc. [11]. In addition, it is evident that some pit defects appear in Figure 4, which may be caused by “target poisoning” or residual stress during the coating deposition [26,27]. As *t*_0_ increases, the number of pit defects decreases. EDS analysis shows that the Ti and C elements in the Ti-DLC coatings are different from the Al, Cu, Si and Ti elements in the pit, and the Al, Cu and Si elements are derived from the Al alloy substrate, as shown in Figure 4 (Spectrum 1–3). It is preliminarily estimated that residual stress is the main factor in the formation of pit defects. That is, the accumulation of residual stress causes spallation of the coating during deposition, thereby forming pit defects.

### 3.2. Composition and Structure Analysis of Ti-DLC Coating

**Figure 5:**
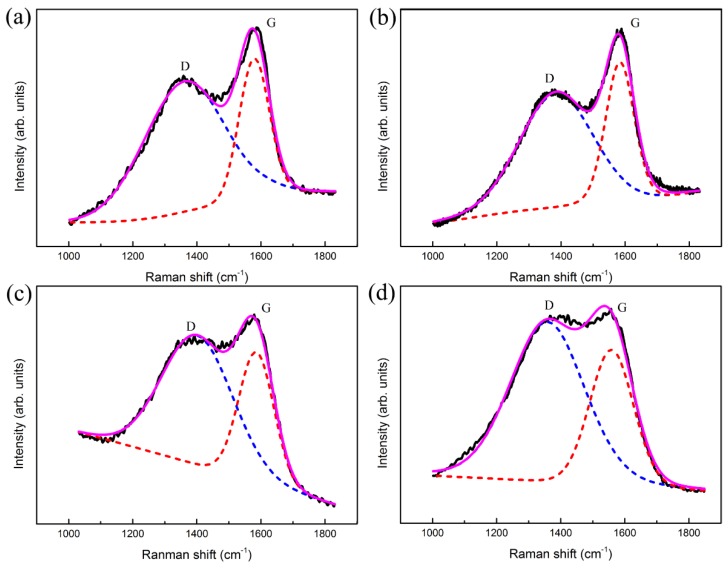
Deconvoluted Raman spectra of Ti-DLC coatings with Ti transition layer thickness (*t*_0_): (**a**) 0 μm, (**b**) 0.5 μm, (**c**) 1.1 μm and (**d**) 2.8 μm.

**Figure 6:**
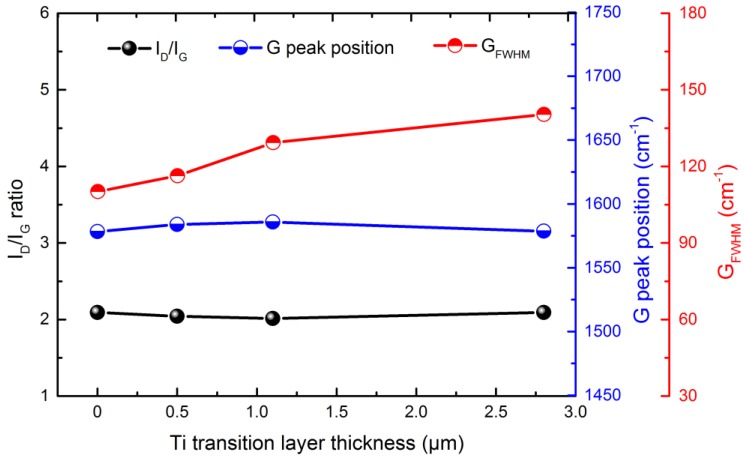
Corresponding I_D_/I_G_ ratio, G peak position and G_FWHM_ of Ti-DLC coatings with Ti transition layer thickness (*t*_0_): (**a**) 0 μm, (**b**) 0.5 μm, (**c**) 1.1 μm and (**d**) 2.8 μm.

Raman spectroscopy and XPS are used to study the chemical bonding state of the elements of the coating, which are the most common methods for investigating DLC. Figure 5 presents the Raman spectra of the Ti-DLC coatings with various *t*_0_ on Al alloys. For all samples, the asymmetric broad peaks appear in the range of 1000–2000 cm^−1^, representing hydrogenated amorphous carbon. A typical DLC Raman spectrum consists of D and G peaks. The Gaussian function was carried out to decompose the Raman spectrum into D and G peaks. All curves show that the D peak is located around 1360–1380 cm^−1^ originating from the symmetric breathing vibration of sp^2^ atoms only in aromatic rings, while the G peak is located around 1560–1580 cm^−1^ originating from the stretching of sp^2^ atoms in both aromatic rings and chains [28]. It is well known that the I_D_/I_G_ ratio (intensity ratio of D peak to G peak), G_FWHM_ (full width at half maximum of G peak) and G peak position can characterize DLC structure, which are related to the grain size and disordered structure [29,30]. As can be clearly seen from Figure 6, the I_D_/I_G_ value is about 2.05, and there is no significant change with the increase of *t*_0_, but there is a significant increase in G_FWHM_. The rise in G_FWHM_ demonstrates an increase in the angle and length distortion of the sp^2^-C bond. In addition, as *t*_0_ increases from 0 to 1.1 μm, it is noted that the G peak position shifts to a higher wavenumber, and then when *t*_0_ further increases to 2.8 μm, the G peak position slightly shifts to a lower wavenumber. The reason for shifting the G peak position from a low wavenumber to a higher wavenumber is the presence of compressive stress in the Ti-DLC coating. These results are consistent with those reported by Pardo et al. and Long et al. [31,32].

**Figure 7:**
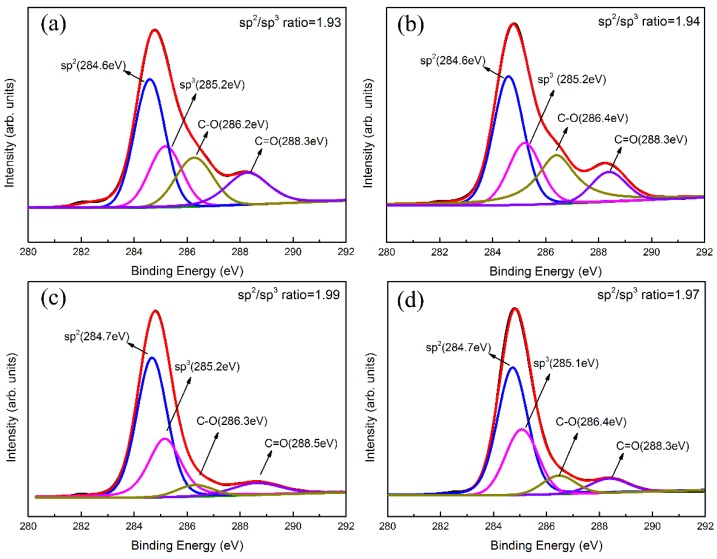
C1s XPS spectra fitting results and sp^2^/sp^3^ ratio of Ti-DLC coatings with Ti transition layer thickness (*t*_0_): (**a**) 0 μm, (**b**) 0.5 μm, (**c**) 1.1 μm and (**d**) 2.8 μm.

**Figure 8:**
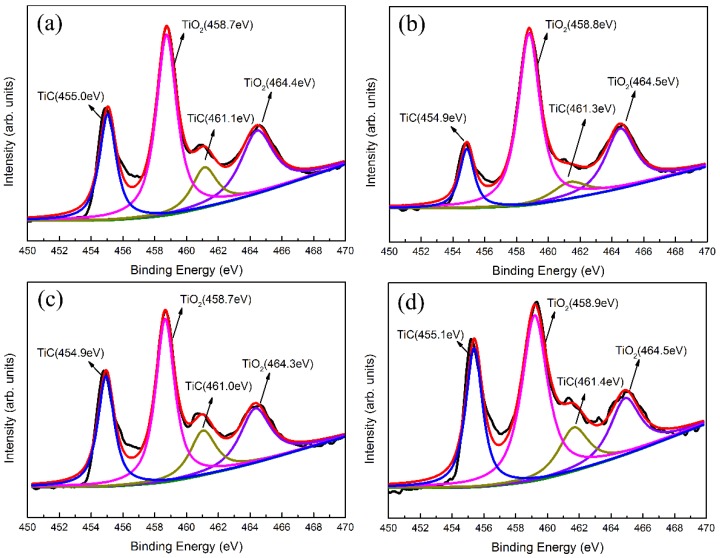
Ti2p XPS spectra fitting results of Ti-DLC coatings with Ti transition layer thickness (*t*_0_): (**a**) 0 μm, (**b**) 0.5 μm, (**c**) 1.1 μm and (**d**) 2.8 μm.

Typical XPS spectra of Ti-DLC coatings with various *t*_0_ are shown in Figure 7 and Figure 8. The C1s spectrum (Figure 7) present, before Ar^+^ etching, a main broad peak around 284.3–285.3 eV. The C1s peak around 284.4 eV originates from the graphitic structure (100% sp^2^) and that for the diamond structure (100% sp^3^), the binding energy is about 285.0 eV, as reported by Lesiak et al. [33]. In order to gain the attribution of each carbon configuration, the C1s spectra are further fitted by a Gaussian function after subtracting the inelastic background. The results show that the C1s core-level spectrum includes four peaks for all samples, corresponding to sp^2^–C bond of 284.6 ± 0.2 eV, sp^3^–C bond of 285.2 ± 0.2 eV, C–O of 286.4 ± 0.2 eV, and C=O of 288.3 ± 0.2 eV, respectively. The C–O and C=O peaks are attributed to impurities from the deposition chamber, which is evident as the chamber pressure has just decreased until the pressure is 4 × 10^−4^ Pa, where there is still a sufficient amount of O_2_. Furthermore, the DLC layer itself is active to air/O_2_ from the chamber and forming O-compounds on the surface. The sp^2^/sp^3^ ratio calculated from the C–C sp^2^ peak area and the C–C sp^3^ peak area [34] is around 1.9, and there is no significant change as *t*_0_ increases from 0 to 2.8 μm, which agrees with the Raman analysis.

Figure 8 shows the XPS spectra of Ti2p with Ti-DLC coatings of various *t*_0_. After deconvolution, all Ti2p spectra present four main peaks, corresponding to two doublet peaks Ti2p_1/2_ and Ti2p_3/2_. The metallic Ti peak (Ti2p_3/2_, 453.8eV) did not appear in these spectra, which proves that all Ti particles are bonded with other species in the coating. These prove that when Ti is doped into the DLC coating, it mainly exists in the form of two titanium compounds [35]. Peaks of 454.9 ± 0.2 eV and 461.2 ± 0.2 eV can be identified as Ti2p_3/2_ and Ti2p_1/2_ of titanium carbide, respectively. The binding energy Ti2p_3/2_ of TiC is located in the range of around 454.6–455.5 eV [23,35]. The other two peaks around 458.7 ± 0.2 eV and 464.5 ± 0.2 eV correspond to Ti2p_3/2_ and Ti2p_1/2_ of TiO_2_, respectively, which proves the presence of oxygen in Figure 7. When Ti is exposed to oxygen in the deposition chamber, titanium oxides are readily formed during the preparation process due to the high reactivity of Ti towards oxygen [36,37,38]. In addition, the binding energy of Ti2p_1/2_ and Ti2p_3/2_ peaks of TiC differs by 6.3 ± 0.2 eV, and the binding energy of Ti2p_1/2_ and Ti2p_3/2_ peaks of TiO_2_ differs by 5.8 ± 0.2 eV. These results are consistent with the values reported in literature [39]. In view of these results, it is found that the Ti state and the binding energy are not related to *t*_0__._

### 3.3. Effect of Ti Transition Layer Thickness on Mechanical Properties of Ti-DLC Coating

**Figure 9:**
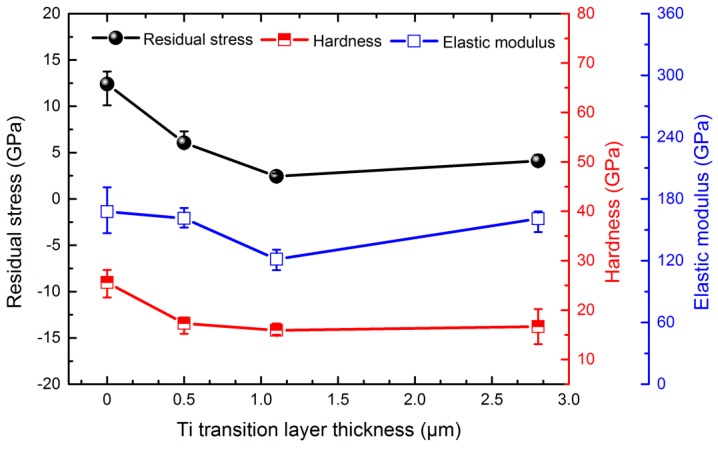
Residual stress, hardness and elastic modulus of Ti-DLC coatings with various Ti transition layer thicknesses (*t*_0_).

The residual compressive stress of the Ti-DLC coatings as a function of the Ti transition layer thickness *t*_0_ (μm) is presented in Figure 9. It can be observed that when *t*_0_ increases, the residual compressive stress decreases sharply from 12.4 GPa at 0 μm to 2.4 GPa at 1.1 μm, which serves as evidence that the residual compressive stress causes the G peak shifting upward to a higher wavenumber as in previous Raman studies [40]. As we know, this strain is produced by different shrinkage tendencies owing to the direct contact between the coating and Al alloy substrate. The Ti transition layer acts as a buffering action on strain, which is the reason of the decrease in residual compressive stress. However, as *t*_0_ increases continuously, the residual compressive stress slowly increases, which may be the consequence of an increase in thermal stress in the coating. The results show that suitably changing the Ti transition layer thickness can effectively adjust residual compressive stress of coatings, which agrees with the report of Liu et al. [25]. Moreover, we found that as the value of *t*_0_ increases, the measured values of hardness and elastic modulus exhibit a decreasing tendency. When *t*_0_ increases from 0 to 1.1 μm, the hardness value decreases from 25 GPa to 16 GPa, and the elastic modulus value gradually decreases from the maximum value of 168 GPa to the minimum value of 122 GPa. Then, when *t*_0_ further increases to 2.8 μm, the hardness value does not significantly change, and the elastic modulus value of the coating increases to 160 GPa. According to the results analysis, the hardness and elastic modulus are dependent on the Ti transition layer thickness. 

### 3.4. Effect of Ti Transition Layer Thickness on Adhesion of Ti-DLC Coating

The adhesion of Ti-DLC coatings on the surface of Al alloys is both an essential property and parameter to determine their service life in engineering applications. The critical load Lc causing an abrupt change in the acoustic signal detection process is used to evaluate the adhesion of the coating by the scratch test. In general, the applied load corresponding to the micro-crack present on the coating is a low critical load defined as Lc1, and the applied load making the coating delaminate from the substrate is a high critical load defined as Lc2. The low Lc1 is difficult to identify during scratch testing [12]. In this regard, the critical load Lc2 is used to evaluate the adhesion of the coating. In order to further characterize the adhesion of the Ti-DLC coating, the appearance of scratches was observed and analyzed by SEM.

**Figure 10:**
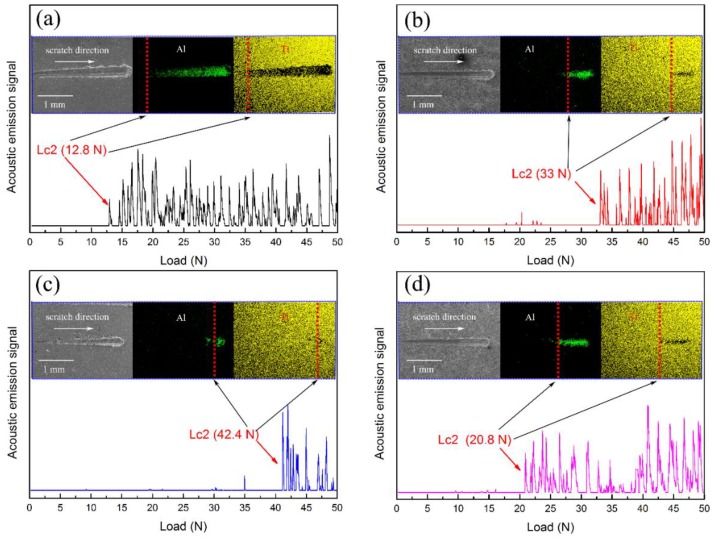
Scratch curves of acoustic signal, SEM images of scratch track and critical load values (Lc2) of Ti-DLC coatings with the Ti transition layer thickness (*t*_0_): (**a**) 0 μm, (**b**) 0.5 μm, (**c**) 1.1 μm and (**d**) 2.8 μm.

Figure 10 presents the acoustic signal of scratch curves, SEM images of scratch track and critical load values (Lc2) for Ti-DLC coatings with various Ti transition layer thickness. The critical loads are 12.8, 33, 42.4 and 20.8 N, corresponding to the Ti-DLC coatings with *t*_0_ of 0, 0.5, 1.1 and 2.8 μm, respectively. The results show that the critical load rises initially from 12.8 N to a maximum of 42.4 N as *t*_0_ increases from 0 to 1.1 μm, and then gradually decreases to 20.8 N at *t*_0_ = 2.8 μm. The failure of the coating is caused by compressive stress which produces a compressive shear fracture of the coating and subsequent delamination during the scratch testing [41]. The Ti transition layer in the Ti-DLC coating allows rectification of the high residual compress stress. Therefore, it can be proposed that the Ti transition layer delays the coating failure, which is why the Ti-DLC coating with the Ti transition layer has a higher critical load as measured in these tests. Among them, 1.1 μm is the optimum Ti transition layer thickness, which is surely favorable for acquiring excellent adhesion between the Ti-DLC coating and the Al alloy substrate.

## 4. Conclusions

Ti-DLC coatings with the different Ti transition layer thicknesses were prepared using FCVA technology. The effect of the Ti transition layer thickness on the structure, mechanical and adhesion properties of the Ti-DLC coatings was systematically investigated. The SEM results illustrate that the Ti-DLC coating possesses a dense composite structure of Ti transition layer/Ti-DLC layer/Ti buffer layer/Ti-DLC layer from the substrate to the top of the coating. The Ti transition layer can improve the interfacial transition, which is beneficial for getting excellent adhesion between the coating and the substrate. Raman and XPS results reveal that the Ti transition layer thickness has no significant influence on the composition and structure of the coating, whereas it affects distortion in the angle and length of the sp^2^–C bond of the coating. The bonding structure of Ti in all coatings exhibits TiC and TiO_2_ phase. The mechanical and adhesion properties analysis demonstrates that the Ti transition layer is favorable to reduce the residual compressive stress of the coating. An increase of the Ti transition layer thickness results in the reduction of the hardness value of the coating. However, its elastic modulus and adhesion exhibit an initial decrease followed by an increasing fluctuation. Moreover, in our study, the Ti-DLC coating has a Ti transition layer thickness of 1.1 μm, exhibiting a minimum residual compressive stress of 2.4 GPa and an optimum adhesion of 42.4 N. This paper outlines an approach to further optimize the adhesion between Ti-DLC coatings and Al alloy substrates, which will provide potential for the widespread application of DLC coatings in Al alloys.

## Figures and Tables

**Table 1:** The *t*_0_ and *t_f_* for the thicknesses of the Ti transition layer and Ti-DLC (diamond-like carbon) coatings with various Ti transition layer deposition time.

Sample No.	*T* (min)	*t*_0_ (μm)	*t_f_* (μm)
1	0	0	5.3 ± 0.62
2	5	0.5 ± 0.15	9.1 ± 0.58
3	10	1.1 ± 0.19	9.5 ± 0.73
4	20	2.8 ± 0.17	10.1 ± 0.62
